# The diagnosis and management of multiple ground-glass nodules in the lung

**DOI:** 10.1186/s40001-024-01904-6

**Published:** 2024-06-01

**Authors:** Quanqing Li, Tianjiao Xiao, Jindong Li, Yan Niu, Guangxin Zhang

**Affiliations:** 1 Department of Thoracic Surgery, Second Hospital of Jilin University, Changchun, Jilin Province, China; 2 Jilin University, Changchun, Jilin Province, China

## Abstract

The prevalence of low-dose CT (LDCT) in lung cancer screening has gradually increased, and more and more lung ground glass nodules (GGNs) have been detected. So far, a consensus has been reached on the treatment of single pulmonary ground glass nodules, and there have been many guidelines that can be widely accepted. However, at present, more than half of the patients have more than one nodule when pulmonary ground glass nodules are found, which means that different treatment methods for nodules may have different effects on the prognosis or quality of life of patients. This article reviews the research progress in the diagnosis and treatment strategies of pulmonary multiple lesions manifested as GGNs.

## Introduction

The detection rate of pulmonary multiple ground-glass nodules in the current clinical diagnosis and treatment process is getting higher and higher. Lung cancer screening by low-dose computed tomograph found that more than half of patients with pulmonary nodules have more than one pulmonary nodule [1, 2]. At present, there is still no clear expert consensus and guidelines for the diagnosis and treatment of multiple ground glass nodules in both lungs. This review focuses on the evaluation of patients with multiple ground glass nodules. This review does not discuss the treatment of single nodules. Because the treatment of multiple ground glass nodules is different, the treatment of the latter is more diverse. This article reviews the definition and epidemiology of multiple pulmonary ground glass nodules. This article also describes the follow-up strategies and treatment options for pulmonary nodules with different shapes and sizes of multiple ground glass nodules.

## Methods

We conducted a literature search of databases such as PubMed / MEDLINE / CNKI to determine that all articles reporting GGN were retrieved. The following terms were used: GGO, multiple pulmonary nodules, GGN, ground glass nodules, partial solid nodules and subsolid nodules, smoking, smoking history.

## Definition and epidemiology

The term GGNS of the lung refers to pulmonary nodules that necessitate thin CT slices (equal to or less than 1.5 mm) acquired during full inspiration, preferably reconstructed using high spatial frequency algorithms, and displayed with a wide window [3, 4], The CT lung window reveals focal areas of increased density, without any discernible evidence of obstruction in the surrounding structures such as vessels and bronchi [5]. Multiple primary lung nodules (MPLN) refer to the concurrent presence of two or more lesions in the lungs, with a diameter ≤ 3 cm, exhibiting multiple ground-glass opacities on high-resolution computed tomography (HRCT). The diagnosis of multiple pulmonary GGN is increasingly common, with approximately 20–30% of resected GGN lesions being accompanied by smaller intrapulmonary GGN lesions [6]. The GGNs were categorized into two subtypes based on imaging findings: pure GGN (pGGN) and mixed GGN (mGGN). According to the results of a randomized controlled trial, a total of 264 pulmonary nodules were detected in 234 out of 7135 volunteers (3.3% prevalence), with multiple pulmonary ground-glass opacity (GGO) nodules observed in 30 participants (12.8%) [7]. Among the 233 patients enrolled in an Early Lung Cancer Action Project (ELCAP) study, 74 individuals (31.7%) exhibited multiple ground-glass nodules [8]. A study demonstrated that CT imaging revealed multiple ground-glass nodules in approximately 25% of patients who underwent surgery for pulmonary GGO nodules [6]. The prevalence of multiple pulmonary nodules in China ranges from 13.26% to 45.56%, with ground-glass nodules (GGNs) accounting for approximately 20% to 40.5% of these cases [9, 10]. According to the available epidemiological data, the prevalence of patients with multiple ground glass nodules accounts for approximately 10–40% of the overall proportion of individuals presenting with such nodules. Currently, there is a relative scarcity of large-scale epidemiological information regarding pulmonary ground glass nodules. As low-dose computed tomography (LDCT) gradually becomes integrated into clinical practice, it is anticipated that more definitive epidemiological findings pertaining to multiple pulmonary ground glass nodules will emerge. Furthermore, the presence of pulmonary nodules is frequently observed among individuals who engage in smoking behavior [11], including individuals presenting with ground glass opacities [12]. Research findings indicate that smoking cessation in patients is associated with a reduction in the size of lung nodules, potentially attributed to smoke-induced inflammation [13]. However, a persistent smoking habit can induce the transformation of lung nodules into malignant tumors, while the diverse pro-cancer molecules present in tobacco smoke may expedite the progression of these nodules [14]. Numerous current studies have consistently demonstrated a strong association between smoking and pulmonary nodules, as the carcinogenic components in smoke exert consistent effects on lung tissue. This may also elucidate why multiple ground glass nodules do not manifest at a single location, suggesting that smoking could potentially contribute to the development of multiple ground glass nodules. However, further validation through extensive epidemiological data is required to establish the precise relationship between smoking and multiple ground glass nodules.

## Relationship between the morphology and size of pulmonary nodules and malignancy and follow-up strategy

### Multiple pure ground glass nodules

The treatment of single-site pulmonary pGGN is already governed by well-defined guidelines [15–18]. For smaller pulmonary ground-glass nodules (< 6 mm), excessive follow-up is currently unnecessary; instead, annual or individualized follow-up intervals are recommended [16], For larger pGGN (> 6 mm), follow-up scans are recommended at 6–12 months, while a safe follow-up period of 5 years is advised. Even if the final pathological diagnosis reveals adenocarcinoma or a precancerous lesion, a follow-up duration of 3–4 years remains safe [16, 19, 20]. For pure ground-glass nodules, a relatively conservative overall treatment strategy is recommended, with regular follow-up being necessary, particularly for larger lesions or those exhibiting signs of progression (such as lesion size, solid part size and proportion, margin and boundary characteristics, and pleural retraction) [21, 22]. Therefore, for multiple pure ground-glass nodules, the follow-up strategy of single pGGN should also be referred to, mainly focusing on the larger ground-glass nodules and the morphology of the nodules, and the possibility of infection factors must be considered. In addition, the risk factors for cancer such as gender, age, family history, previous medical history, tobacco and other carcinogens inhalation history should also be considered comprehensively.

### Partially solid ground-glass nodules

Yankelevitz et al. found 2392 (4.2%) pure ground-glass (nonsolid) nodules in a large screening study of 57,496 baseline studies. Of these nodules, a total of 73 subsequently proved to be adenocarcinoma. Of these, 19 (26%) malignant nodules had solid components from pure ground-glass nodules [23]. The characteristics of ground glass nodules with increased solid components were compared to those of unchanged GGNs in a study aimed at identifying factors associated with GGN growth. The findings revealed that only partially solid ground glass nodules exhibited a significant association with nodule growth (*p* < 0.001), while no other factors demonstrated an association with increased size [24]. Moreover, the contribution of ground-glass nodules components in patients with aggressive stage I non-small cell lung cancer (NSCLC) was elucidated. Oncologic outcomes were assessed based on the presence of GGN components, revealing a significant disparity in 5-year recurrence-free survival rate (100% for pure GGN group versus 87.6% for partial solid group) [25]. The statistical analysis of 42 surgically diagnosed patients with bronchioloalveolar carcinoma (BAC) or adenocarcinoma revealed a significant correlation between the proportion of solid components in mGGN pulmonary lesions and the likelihood of matrix invasion [26]. This demonstrates that the presence or increase of solid components not only contributes to the growth of ground glass nodules but also impacts their prognosis, making it a crucial consideration in clinical decision-making. Therefore, for partially solid ground glass nodules or pure ground glass nodules, greater attention should be devoted to monitoring changes in solid components. In summary, when determining the treatment strategy for multiple ground glass nodules, priority should be given to assessing the solid components of these nodules. If there are any nodules with solid components among multiple GGNs, follow-up should primarily focus on these rather than slightly larger pure ground glass nodules.

## Selection of treatment options (diagnosis and therapeutic interventions)

For solitary ground-glass nodules, a wide range of treatment modalities are available. Depending on the anatomical location and size of the GGN, various approaches can be considered including CT-guided percutaneous pulmonary nodule biopsy, surgical interventions (such as sublobar resection or lobectomy), and tumor interventional ablation techniques, The following treatments are frequently employed in clinical practice.

### CT-guided biopsy

CT-guided biopsy is not recommended for isolated pure ground-glass nodules, as per the guidelines [17, 18, 27]. It is caused by the absence of solid components, the false-negative rate is elevated, and other complications may also impact the diagnostic judgment. In a study involving 28 patients with ground-glass nodules (GGN) lesions who underwent CT-guided needle biopsy, it was observed that the diagnostic accuracy of needle aspiration biopsy in pure GGN lesions was significantly lower compared to mixed GGN lesions. The sensitivity, specificity, and accuracy for diagnosing GGN based on its components were found to be 50%, 100%, and 57% in mixed GGN lesions, respectively; while in partially solid ground-glass nodules lesions they were 82%, 100%, and 90%, respectively [28]. A retrospective analysis involving 55 cases of ground-glass nodules demonstrated that pure GGN lesions on CT were more likely to be diagnosed as simple BAC, while GGN-dominant lesions were more likely to be identified as adenocarcinoma with interstitial invasion. In a study comprising 73 patients with 85 biopsies, the sensitivity, specificity, and accuracy for diagnosing GGN based on composition were found to be 68.4%, 100.0%, and 73.9% for pure ground-glass nodules; 100.0%, 100.0%, and 100.0% for GGN lesions with solid components ≤ 5 mm; and finally, 92.9%, 100.0%, and 94.9% for GGN lesions with solid components > 5 mm in size, respectively [29]. This clearly demonstrates that the accuracy of biopsy is influenced by the presence of solid components, thus indicating the potential for performing biopsies to confirm pathology in cases of mGGN lesions. In instances of multiple ground glass nodules, if no complications arise during the puncture procedure, it is advisable to conduct multiple puncture biopsies on a single large mGGN or multiple mGGN to enhance the likelihood of obtaining positive results and avoid false negatives, thereby facilitating improved treatment decision-making for patients with multiple ground glass nodules. However, it is important to note that an increase in the number of punctures may lead to a gradual rise in associated complications.

### Surgical treatment

There exist well-established guidelines for the surgical management of ground-glass nodules, with sublobar resection (segmentectomy or wedge resection) or lobectomy being viable options. However, recent research has shown a growing preference for sublobar resection in cases of small solitary pulmonary nodules. Nevertheless, the choice of surgical approach should also consider factors such as nodule number and location, solid component diameter to maximum nodule diameter ratio (CTR), and the patient's underlying cardiopulmonary function. Sublobar resection offers advantages over lobectomy including enhanced preservation of pulmonary function, reduced incidence of surgical complications, decreased intraoperative blood loss, shorter thoracic drainage time, and a shorter hospital stay [30–32]. The incidence of multiple primary lung ground-glass nodules exhibited a gradual increase during the same period, indicating a pathological manifestation of multiple primary lung cancer [33, 34]. Multiple ground-glass nodules can be observed in the lung, including GGNs within the same lobe of the ipsilateral chest, GGNs across different lobes of the ipsilateral chest, and GGNs present bilaterally involving multiple chests and lobes.

- a. Multiple ground-glass nodule were observed in the ipsilateral thoracic cavity and within the same lobe

In cases where multiple nodules are localized within the same lobe, with a small number of nodules and concentrated distribution, the preferred surgical approach would be wedge resection or segmentectomy. However, in situations where there is a large number of scattered nodules within the same lobe, lobectomy should be considered. In instances with adequate preoperative planning, combined subsegmentectomy may also be contemplated.

- b. Multiple ground-glass nodules (GGNs) were observed in different lobes of the ipsilateral thoracic cavity

A study demonstrated a significantly higher incidence of malignancy in ipsilateral nodules removed during the primary lesion surgery (39%) compared to those observed during long-term follow-up (4.8%) [35]. Furthermore, the comprehensive excision of all lesions in a single surgical procedure not only alleviates postoperative anxiety among patients but also minimizes the potential for complications and risks associated with subsequent surgeries. Therefore, it is advisable to remove all multiple ground-glass nodules located in different lobes of the same side of the thoracic cavity during one operation whenever feasible. Naturally, further prospective experiments are still required to validate its accuracy.

- c. Multiple ground-glass nodules are observed in the bilateral thoracic and pulmonary lobes

Given the intricacies of thoracic surgery, unilateral procedures have traditionally been favored in clinical practice; however, recent evidence challenges this notion. A study involving 151 patients demonstrated that for individuals without relevant risk factors, bilateral surgeries do not result in increased severity or complications [36], there is a greater body of literature available to substantiate this conclusion [37, 38]. However, further controlled trials are still required to substantiate this conclusion. Therefore, for patients with bilateral multiple ground-glass nodules and good physical condition (without evident underlying diseases), simultaneous bilateral surgery is recommended to maximize the removal of all pulmonary nodules. For patients who are deemed unsuitable for simultaneous bilateral surgery after evaluation, the primary lesion should be excised based on its size and changes in solid components, while avoiding lobectomy to preserve more healthy lung tissue.

In conclusion, for patients with multiple pulmonary ground-glass nodules, whenever feasible based on the patient's physical condition, it is recommended to surgically remove all GGN lesions as extensively as possible. For cases where complete resection is not achievable, a combination of interventional ablation and other treatment modalities should be considered.

### Percutaneous interventional ablation therapy

Currently, advancements in CT technology have led to the development of novel surgical approaches for managing multiple pulmonary nodules, such as percutaneous interventional therapy for lung cancer. This therapeutic modality primarily encompasses thermal ablation techniques including radiofrequency ablation (RFA), microwave ablation (MWA), cryoablation, etc. In order to assess the efficacy of percutaneous interventional ablation in treating multiple ground glass nodules bilaterally, it is imperative to establish survival indices. A clinical trial involving 42 GGNs with over 50% ground glass content demonstrated tumor-specific survival rates of 100% at 1, 3 and 5 years, respectively, without any procedure-related mortalities; however, complications were observed in 28.6% of cases [39]. The data presented herein suggested that microwave ablation is a safe and viable treatment option in the short term for patients with unresectable pulmonary nodules [40, 41]. However, there is still a dearth of long-term follow-up data. Furthermore, multiple studies lend support to this conclusion. In the diagnosis of lung ground-glass nodules, numerous clinical data indicate the potential presence of lymph node metastasis. A study encompassing 867 patients with GGN-type lung cancer revealed that approximately 2.9% of patients experienced lymph node metastasis, with rates of lymph node metastasis for pGGN, mGGN with CTR < 0.5, and mGGN with CTR > 0.5 being 0%, 6.9%, and 9.1%, respectively [42]. A retrospective analysis revealed that among 55 cases of pGGN, no lymph node metastasis was observed, whereas out of 292 cases of mGGN, 9 exhibited lymph node metastasis [43]. Based on extensive research data, it has been observed that pGGN exhibits no evidence of lymph node metastasis, while lesions with solid components comprising more than 25% demonstrate a significantly elevated risk of lymph node involvement [42, 44–46]. Therefore, percutaneous interventional therapy offers distinct advantages and disadvantages. The benefits include minimal trauma, high safety profile, low economic burden, limited lung tissue injury, negligible impact on lung function, ability to perform multiple operations for multiple nodules, and synchronous biopsy for patients without pathological diagnosis. However, there are limitations such as significant individual variations in ablation parameters and stringent requirements for lesion location determination. In addition, the inability to clear lymph nodes hinders the determination of subsequent treatment options.

In summary, when selecting candidates for percutaneous interventional ablation procedures that cannot biopsy lymph nodes or nodules with a low proportion of solid components or low likelihood of lymph node metastasis should be prioritized whenever possible. Conversely, if a ground-glass nodule exhibits a high proportion of solid components and the patient's physical condition permits it, surgical treatment is recommended.

## Progress and feasibility of targeted therapy

Pulmonary ground-glass nodules are an indeterminate finding that have been associated with benign inflammatory diseases, fibrosis, atypical adenomatous hyperplasia, bronchioloalveolar carcinoma (BAC or adenocarcinoma in situ), and BAC-dominant adenocarcinoma (microinvasive adenocarcinoma) [47–50]. Pulmonary nodules exhibiting a ground-glass appearance predominantly represent bronchioloalveolar carcinoma and precancerous lesions. The evolutionary trajectory of these nodules progresses from pure ground-glass nodules to mixed ground-glass nodules, ultimately culminating in solid nodules. Deciphering the driving factors underlying this process is of paramount importance as they hold potential diagnostic and therapeutic implications. Distinct gene mutations exhibit diverse biological behaviors, with studies demonstrating that EGFR and KRAS mutations are associated with aggressive and rapid growth of these nodules [51–54]. EGFR mutations represent the most prevalent and primary driving force behind early disease progression [51, 55]. Nodules harboring BRAF, ERBB2, and MAPK2P1 mutations exhibit an indolent behavior and a higher likelihood of being classified as part-solid ground-glass nodules [51], The absence of EGFR, KRAS, ALK and HER2 mutations was found to be associated with no growth in GGN. In addition, this study revealed a high incidence of EGFR mutations and a low incidence of KRAS mutations, which may be attributed to ethnic characteristics and other factors [54]. Several studies have demonstrated a correlation between EML4-ALK and ROS1 mutations and the aggressive nature of ground-glass nodules [56, 57]. Further exploration of molecular mechanisms is warranted. Research has demonstrated that tumor heterogeneity, characterized by the presence of distinct cancer clonality within the same patient, resulting from anatomical selection or temporal evolution, can serve as a predictive factor for the patient's response to targeted therapy [58]. The heterogeneity between primary tumors and metastatic tumors is widely recognized as the most prevalent form of tumor heterogeneity. This phenomenon also extends to multiple primary lung cancers with multiple ground-glass nodules (GGNs), posing significant challenges to the advancement of precision medicine. Therefore, deciphering this heterogeneity holds immense significance, as it has the potential to enhance our comprehension of cancer biology, encompassing its genome, epigenome, functional diversity, and mechanisms underlying treatment resistance [59]. A study demonstrated that NSCLC patients with multiple GGNs exhibited EGFR mutations, with a remarkably high inconsistency rate of 70.8% (17 out of 24 cases), thereby indicating significant tumor heterogeneity [56]. The presence of multiple GGNs in NSCLC patients suggests their origin from distinct primary clonal sources [56, 60, 61]. This implies that each nodule in multiple pulmonary ground-glass nodules (GGNs) is autonomous and necessitates distinct treatment approaches from intrapulmonary metastasis, thereby posing challenges to personalized therapy. Studies have revealed that while mutations within the same nodule are mutually exclusive, multiple signaling pathways can concurrently be shared among multiple nodules in the same patient. For instance, even within EGFR mutations, various subtypes of EGFR mutations can still be shared by multiple nodules [62]. Therefore, it is imperative to perform independent gene and molecular detection of each GGN nodule for targeted therapy. The advancements in gene sequencing technology, particularly single-cell sequencing and whole genome sequencing, have continuously unveiled the diversity of gene mutations at the cellular and molecular levels in GGNs, thereby facilitating progress in targeted therapy. Currently, apart from surgery, there are no safe, accurate, and noninvasive methods available to determine the mutation status of tumors with multiple GGNs. Henceforth, it is crucial to further develop noninvasive and highly sensitive approaches to advance novel therapies like targeted therapy for GGNs.

## Conclusions

With the advancement of LDCT, an increasing number of multiple ground-glass nodules have been detected in both lungs. While the diagnosis and treatment of early lung cancer with a single GGN has become relatively mature, there is currently no clear consensus on the optimal diagnostic and treatment strategies for multiple GGNs. This article provides a comprehensive review of current progress in this area.

## Data Availability

No applicable.

## Abbreviations

LDCT: Low-dose computed tomography
GGNs: Ground glass nodules
GGO: Ground-glass opacity
MPLN: Multiple primary lung nodules
HRCT: High-resolution computed tomography
CT: Computerized tomography
pGGN: Pure ground glass nodule
mGGN: Mixed ground glass nodule
